# The Use of Veratrum Viride as an Arterial Sedative, First Discovered by a Veterinary Surgeon

**Published:** 1862-02

**Authors:** 


ARTICLE VII.

THE DISCOVERY OF THE USE OF

### VERATRUM VIRIDE AS AN ARTERIAL SEDATIVE,

FIRST MADE BY A VETERINARY SURGEON.

It is well known that some fifty years ago, veratrum viride was experimented upon by physicians in New England, and abandoned, because it was supposed to be a harsh, unmanageable narcoctic and emetic. A few years since, it was brought anew into notice by Dr. Norwood, of North Carolina, who proved that when properly used, it was a pure cardiac sedative, of unerring certainty in its action, and capable of substituting itself for the lancet, in great numbers of inflammatory cases.

I have been interested, lately, in ascertaining that twenty years ago, the same discovery was made, and constantly made use of by a veterinary surgeon in Central New York, but as he unfortunately, did not publish his observations, nor extend them beyond the treatment of brute animals, the human species, less favored than the horses, did not, for many years, get the benefit of the remedy.

Alpheus Rawson, of Weedsport, Cayuga Co., N. Y., having to deal extensively in horses, took to the study of their diseases, to protect himself against loss. He read all the books obtainable on the subject, and pursued equine dissections with avidity, and finally became noted as a curer of equine diseases. In his earlier practice, he used venesection very freely whenever a hard frequent pulse showed its necessity; to use his own expression, he "carried the lancet in both hands." After some years, he made the discovery that the juice of the American Hellebore would reduce the force and frequency of a horse's pulse, with all the promptness and certainty of the lancet. Accordingly, he began to employ it freely as a substitute, and ultimately, almost ceased to draw blood. He used to send to Connecticut for the veratrum, obtaining a full cask of fresh roots at a time. These he boiled in water, and obtained, by evaporation, a thick watery extract of the consistency of treacle and of great potency. His practice was, to stand by the horse with his hand upon the pulse, and with his finger place a little of the extract upon the animal's tongue. He watched for the effect, and added a little more from time to time, until the pulse was well reduced. He stated, that in the horse, the first effects of the remedy were perceptible in a very few minutes, so that it would seem to act more promptly upon that animal than upon man. His extract, however, must have been of great strength. One drop of an extract as thick as treacle, would be equal to, perhaps, twenty or thirty drops of Norwood's tincture. E. A.

